# Peoria District Medical Society

**Published:** 1847-09

**Authors:** E. Andrew

**Affiliations:** Secretary


*Peoria District Medical Society.*—Pursuant to public notice a number of the regularly qualified practitioners of medicine of Peoria, Tazewell, McLean, Woodford, Putnam, Bureau, Stark, Knox, and Fulton counties, in the state of Illinois, met at the court house in the city of Peoria, on Tuesday the 13th of July, and organized the "Peoria District Medical Society."

The following persons were admitted as members:—Rudolphus Rouse, Peoria; A. G. Henry, Pekin; Francis A. McNiel, Peoria; L. A Hanaford, Trivoli; Edward Dickinson, Peoria; Joseph C. Frye, Peoria; Elwood Andrew, Peoria; Thomas J. Moore, Trivoli; S. Christy, Farmington; G. H. Hickman, Farmington; O. N. Williams, Farmington; Wm. Cromwell, Pekin; M. B. Van Petten, Trivoli; H. H. Sexton, Galesburgh; E. M. Colbourne, Bloomington; Uri P. Golliday, Kickapoo; Alvah Leonard, Kickapoo; John L. Huff, Maquon; Charles Cutter, Princeville; T. P. Rogers, Washington; John Riley, Henderson; Alanson Stockwell, Tremont; E. V. Garfield Toulon; Jerome B. Tenney, Tremont; John Murphey, Peoria; G. P. Wood, Washington; Robt. B. M. Wilson, Peoria; John B. McDowell, Lewiston; Thomas Hall, Toulon; A. H. Lace, Bloomington; N. S. Tucker, Peoria.

The following officers were elected for the ensuing year:

President.—F. A. McNiel, M. D.

Vice Presidents.—A. G. Henry, M. D., J. C. Frye, M. D.

Recording Secretary.—E. Andrew, M. D.

Corresponding Secretary.—Jno. Murphy, M. D.

Treasurer.—E. Dickinson, M. D.

Censors.—Drs. R. Rouse, E. M. Colbourne, T. P. Rogers, H. H. Sexton and Thomas Hall.

The next meeting of the society will be held at the court house, in the city of Peoria, on the first Tuesday of November next, to which all qualified Physicians, desirous of becoming members, are invited to attend. The delegates to the American Medical Association, will be appointed at the November meeting.

An address will be delivered by the President—one or more lectures, by persons appointed for the purpose—after which, the nature, pathology, and treatment of congestive fever, will be discussed by the members of the society.

E. Andrew, Secretary.

